# Lessons Learned from Dengue Surveillance and Research, Puerto Rico, 1899–2013

**DOI:** 10.3201/eid2508.190089

**Published:** 2019-08

**Authors:** Tyler M. Sharp, Kyle R. Ryff, Gilberto A. Santiago, Harold S. Margolis, Stephen H. Waterman

**Affiliations:** Centers for Disease Control and Prevention, San Juan, Puerto Rico, USA (T.M. Sharp, K.R. Ryff, G.A. Santiago, H.S. Margolis, S.H. Waterman);; US Public Health Service, Rockville, Maryland, USA (T.M. Sharp, S.H. Waterman)

**Keywords:** dengue, history, Puerto Rico, surveillance, viruses, research

## Abstract

Dengue was first reported in Puerto Rico in 1899 and sporadically thereafter. Following outbreaks in 1963 and 1969, the Centers for Disease Control and Prevention has worked closely with the Puerto Rico Department of Health to monitor and reduce the public health burden of dengue. During that time, evolving epidemiologic scenarios have provided opportunities to establish, improve, and expand disease surveillance and interventional research projects. These initiatives have enriched the tools available to the global public health community to understand and combat dengue, including diagnostic tests, methods for disease and vector surveillance, and vector control techniques. Our review serves as a guide to organizations seeking to establish dengue surveillance and research programs by highlighting accomplishments, challenges, and lessons learned during more than a century of dengue surveillance and research conducted in Puerto Rico.

In 1916, Walter W. King (Figure 1), a surgeon in the US Public Health Service stationed at the San Juan (Puerto Rico) Quarantine Station, presented to the American Society of Tropical Medicine a firsthand account of his experiences during the 1915 dengue outbreak in Puerto Rico (*1*). Health Commissioner William Lippitt had invited Henry Rose Carter and William Gorgas to work with King, then a captain in the US Army Medical Corps, to determine whether yellow fever virus or dengue virus caused the outbreak. After Carter, who had survived a bout with yellow fever years earlier and thus was immune, fell ill soon after examining patients in a mosquito-infested hospital, the team concluded that dengue caused the outbreak (*2*). King credited Arthur H. Glennan, his predecessor at the San Juan Quarantine Station, as the first to have reported local dengue cases in Puerto Rico in 1899 (*3*). King also cited local physicians who reported having seen dengue cases nearly every year since and an apparent outbreak in 1905 (*1*). King noted that younger persons and residents of San Juan were affected more often than elderly persons and persons from rural areas and that the epidemic was associated with a “superabundance” of *Aedes* mosquitoes. In addition, dengue cases frequently appeared in the same household ≈2 weeks after the first household member fell ill, which suggested to King that infections might occur around the household. These observations collectively led King to suspect that dengue already was endemic in Puerto Rico by 1915.

**Figure 1:**
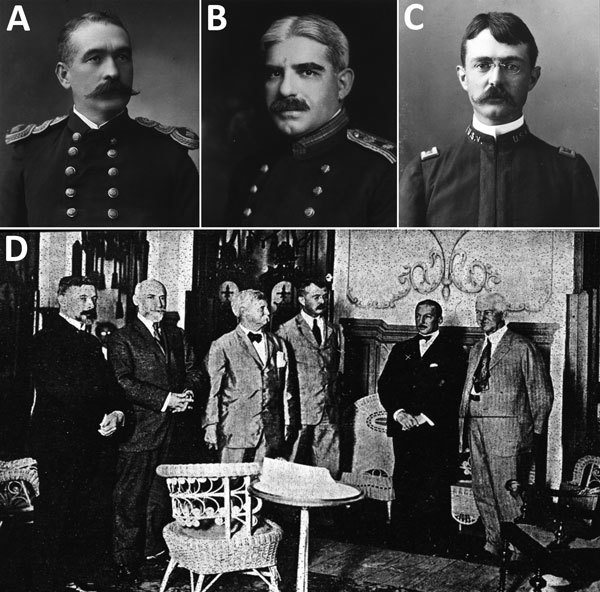
Prominent public health figures in Puerto Rico during the early 1900s. A) Assistant Surgeon General Arthur H. Glennan, pictured circa 1895. B) Walter W. King, Chief Quarantine Officer of the US Quarantine Station, San Juan, Puerto Rico, pictured in 1915. C) San Juan Health Commissioner William F. Lippitt, pictured in 1899. D) From left to right: Puerto Rican tropical medicine physicians Isaac González Martínez and Pedro Gutiérrez Igaravídez met with yellow fever expert Henry Rose Carter, San Juan Commissioner of Health William Lippitt, Mariano Lebredo from Cuba, William Gorgas, and (not pictured) Bailey K. Ashford and Walter W. King to determine the etiology of an outbreak in 1915 that was ultimately attributed to dengue. Images were obtained from the National Library of Medicine (A–C) or were originally published in Puerto Rico Ilustrado (https://en.wikipedia.org/wiki/Puerto_Rico_Ilustrado) (D).

Many of King’s prescient observations remain true. Yet, despite extensive resources expended to understand and combat dengue, rates of illness and death caused by dengue continue to increase worldwide (*4*). We describe lessons learned during >100 years of dengue surveillance and research in Puerto Rico. (Because the names of some entities have changed since 1899, we have used their contemporary names to maintain consistency.)

## Early Epidemiologic Investigations

Only 1 report of dengue in Puerto Rico was published in the nearly 50 years after King’s report (*5*). In 1963, the Puerto Rico Department of Health (PRDH) requested assistance from the Centers for Disease Control and Prevention (CDC) to respond to a dengue outbreak in which ≈27,000 suspected cases were ultimately reported to PRDH by telegram from across the island (Table 1) (*6*,*15*). A team was sent from CDC headquarters to help PRDH respond to the outbreak, along with colleagues from CDC’s Puerto Rico Field Station, which had been established in 1951 to research and control schistosomiasis and investigate rabies, histoplasmosis, and leptospirosis. Later known as the San Juan Laboratories, the Field Station had grown out of the Office of Malaria Control in War Areas, which became CDC in 1946.

**Table 1:** Dengue outbreaks and epidemics, Puerto Rico, 1899–2013*

Year(s)	DENV(s)†	No. reported suspected cases (cases/1,000 pop)	Most affected age group(s), y†	No. reported DHF cases (DHF cases/1,000 dengue cases)	Reported dengue-related deaths (deaths/1,000 dengue cases)	Reference
1899	Unknown	“Some”	NA	NA	NA	(*3*)
1915	Unknown	Hundreds or thousands (≈20)	<10	NA	0 (0)	(*1*)
1963	3	≈27,000 (NA)	20–29, 30–39, 10–19	0	NA	(*6*,*7*)
1969	2	16,665 (NA)	30–49	0	0	(*8*)
1977	2, 3, 1	12,733 (3.75)	15–19, 20–29, 10–14	0	0	(*9*)
1978–1979	1, 2, 3	12,314 (3.63)	15–19, 20–29, 10–14	0	0	‡
1981–1982	1, 4	≈17,160 (NA)	NA	NA	5 (≈0.3)	‡
1986	4, 1, 2	10,659 (NA)	6–15, 31–45, <1	29§ (27§)	3† (0.3)	(*10*)
1994–1995	2, 4, 1	24,700 (7.0)	15–19, 10–14, 20–24	152 (6.2)	40 (1.6)	(*11*)
1998	4, 1, 2, 3	17,000 (4.8)	10–19, <1	174 (10.2)	56 (3.3)	(*12*)
2007	3, 2, 1, 4	10,508 (2.7)	10–14, 15–19, <1	227 (21.6)	40 (3.8)	(*13*)
2010	1, 4, 2, 3	26,766 (7.2)	10–14, 15–19, 5–9	448 (16.7)	128 (4.8)	(*14*)
2012–2013	1, 4, 2, 3	30,921 (8.6)	10–14, 15–19, <1	11 (0.4)	199 (6.4)	‡

*DENV, dengue virus; DHF, dengue hemorrhagic fever; NA, data not available; pop, population. †In order of relative frequency. ‡Centers for Disease Control and Prevention and Puerto Rico Department of Health, unpub. data. §Laboratory-positive cases; number of suspected cases not available.

Through observation of 2,777 persons during the 1963 outbreak, dengue was described as an acute febrile illness lasting 4–7 days with infrequent minor hemorrhagic manifestations (*6*). Two thirds of persons with serologic evidence of infection reported a recent illness consistent with dengue (*6*). Distinct from King’s observations from 1915, in 1963 all age groups were equally affected by both illness and infection, suggesting the outbreak was caused by a virus type that had not previously circulated in Puerto Rico. Further analysis revealed that only persons >25 years of age had serologic evidence of prior exposure to any of the 4 dengue virus types (DENV-1–4), suggesting that a dengue outbreak might have occurred during the late 1930s or early 1940s, consistent with reports of a dengue-like illness of “minor epidemic proportions” in 1945 (*5*).

PRDH again requested assistance from CDC during an outbreak of DENV-2 in 1969 (*8*). Serosurveys in 4 neighborhoods in northern Puerto Rico demonstrated that 47% of participants had been infected with a DENV and that 43% of infections were asymptomatic (*8*). Moreover, investigators found no evidence of protective immunity in persons who reported having been ill during the 1963 epidemic, again demonstrating type-specific immunity. Aerial spraying of malathion was used to combat the epidemic in 1969, but malathion was observed to not efficiently enter households, where most *Aedes* mosquitoes are present, and a natural decline in cases precluded analysis of its effectiveness in reducing transmission (*16*).

## Evolution and Improvement of Case Surveillance

An islandwide case reporting system (later named the Passive Dengue Surveillance System [PDSS]) was established in 1969 to collect basic demographic and clinical data from patients with suspected dengue. By 1970, PDSS enabled detection of dengue cases in southwestern Puerto Rico during the dry season, providing further evidence that dengue was endemic (*17*); however, later reports questioned this finding (*18*). In 1973, CDC’s mission in Puerto Rico included studying dengue, assisting PRDH to operate PDSS, and identifying approaches to combat dengue.

Surveillance in subsequent years demonstrated that detection of cases based solely on clinical signs and symptoms (i.e., syndromic surveillance) was insufficient to monitor dengue because clinicians were often unable to distinguish dengue from influenza, leptospirosis, rubella, and other common causes of acute febrile illness (*9*,*18*). In response, laboratory-based surveillance for dengue was initiated in 1974. Cross-island expressways opened in the mid-1970s, resulting in increased detection of dengue cases from throughout the island because of more rapid dissemination of infections and improved case detection. In 1975, the importance of improving surveillance in small outpatient clinics was identified as a priority to quantify the incidence of dengue in rural communities. After epidemics in 1977 and 1978, in 1981 CDC’s mission in Puerto Rico was officially changed to focus primarily on dengue (Table 2).

**Table 2:** Nomenclature and chiefs of the Centers for Disease Control and Prevention* research station located in San Juan, Puerto Rico, 1951–present

Year	Name	Chief	Location
1951–1954	Communicable Disease Center, Ecologic Investigations Program, Puerto Rico Field Station	David Pimentel	La Puntilla, Arsenal, Viejo San Juan (1951–1972)
1954–1955		Charles S. Gerhardt	
1955–1970		Frederick “Fred” Ferguson	
1970–1976	Center for Disease Control, San Juan (Tropical Disease) Laboratories	Barnett “Barney” Cline	Rio Piedras (1972–2000)
1976–1980		John “Jack” Woodall	
1980–1981		Ernest Ruiz Tibén (interim), Roslyn Q. Robinson	
1981–1989	Centers for Disease Control (and Prevention), Dengue Branch†	Duane J. Gubler	
1989–2006		Gary G. Clark	
2006–2008		Wellington Sun	Puerto Nuevo (2000–present)
2008–2010		Kay M. Tomashek (interim)	
2010–2015		Harold “Hal” S. Margolis	
2015–present		Stephen “Steve” H. Waterman	

*Current name. †Currently in the Division of Vector-Borne Diseases, National Center for Emerging and Zoonotic Infectious Diseases.

In 1982, a new surveillance system that included active surveillance sites to augment passive reporting was implemented; this system emphasized monitoring virus transmission in 31 sites across the island (*19*). Approximately 100 acute serum specimens were processed for virus isolation and serology each week using new diagnostic methods (*19*). Test results were reported to surveillance sites, and close communication was maintained with clinicians. Prompt communication of test results provided a sensitive spatial and early warning system of increased DENV transmission around the island. A monthly surveillance summary was published and sent to stakeholders in Puerto Rico and elsewhere, and in 1987 a computerized system was implemented to track and manage data. Although this surveillance system provided early epidemic warnings, the control program was still reactive instead of proactive, and ultimately response efforts did not appreciably affect epidemic trends.

**Figure 2:**
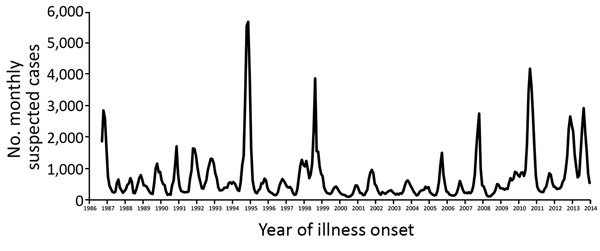
Suspected dengue cases reported to the Puerto Rico Department of Health, by month, 1986–2013. The healthcare system of Puerto Rico changed from public to semiprivate in 1994.

In 1994, the government-funded healthcare system was privatized with the aim of increasing efficiency, streamlining bureaucracy, and decreasing government expenditures. A consequence was that public health nurses and vector control staff, whose regional offices had been in government-operated hospitals that managed ≈60% of all patients, became disconnected from clinicians. This change resulted in a decreased capacity to report suspected dengue cases and disproportionate case reporting from outpatient clinics. In the early 1990s, ≈42% of hospitalized dengue patients were reported (*20*,*21*); after the change in the structure of the healthcare system, this proportion decreased to ≈16% (*22*). As a result, comparing epidemiologic trends from before and after the mid-1990s is difficult (Figure 2).

During the large dengue epidemic of 1994 (*11*), the volume of case report forms and specimens overwhelmed the available capacity for data management and diagnostic testing, resulting in substantial delays in real-time analysis of case data. In part because of this delay, local authorities disputed that an epidemic was in fact occurring (*23*). In response, a standardized method was needed to determine when dengue epidemics were occurring. Epidemiologists at CDC and PRDH developed a method wherein weekly dengue surveillance data were tabulated along with deviation bar charts, which were used to compare data from the municipalities experiencing epidemics with historic averages (*24*). This method enabled a rigorous method to define epidemics, which in turn enabled PRDH to initiate early intervention strategies. Following this approach, the islandwide incidence of dengue cases has been summarized in weekly reports since the early 2000s. When 2 consecutive weeks of above-threshold cases are reported with a concomitant increase in laboratory-positive dengue cases, an epidemic was to be declared and response activities initiated.

## Changes in Disease Severity that Necessitated Innovations in Case Surveillance

After increasing reports of dengue hemorrhagic fever (DHF) in Southeast Asia during the 1970s, increased effort was dedicated to monitoring the clinical severity of dengue in Puerto Rico. The first DHF cases were retrospectively detected from 1975 (*25*), and the first confirmed dengue-related death was identified in 1982 (*26*). DHF cases were again detected in 1985, and increased numbers of DHF and fatal cases were detected during the 1986 epidemic (*10*) and continued into 1987.

Starting in the early 1980s, collaborations were established with regional infectious disease physicians and neurologists to monitor fatal dengue-like illness, and the Demographic Registry of Puerto Rico consulted physicians who had listed dengue on a patient’s death certificate. Because of the time-consuming nature of this process and media claims of unreported fatal dengue cases, PRDH and CDC implemented new approaches to better understand and quantify the incidence of fatal dengue.

First, medical examiners were identified as an ideal resource to detect dengue-related deaths (*27*). CDC, PRDH, and the Puerto Rico Institute of Forensic Sciences also collaborated to collect tissue specimens during autopsy of persons who died after an acute febrile illness. In addition, diagnostic testing using immunohistochemical analysis and PCR enabled the diagnosis of dengue cases that would have been missed by only testing serum (*28*). Last, increasing recognition of DENV as a cause of severe neurologic illness led to enhanced surveillance for neuropathies associated with DENV infection in 2003 and subsequent estimation that dengue with neurologic manifestations was an outcome with comparatively low incidence (*29*).

## Community and Clinician Education Campaigns

During the 1963 epidemic and for years thereafter, outbreak response activities focused on space spraying with insecticides, community cleanup campaigns, and educational activities to inspire community-based vector control campaigns. A major community-based control program supported by Rotary International in collaboration with CDC and PRDH included outreach through school education programs, church and community organizations, and clinicians. A medical anthropologist was hired in 1986 to help CDC, PRDH, and local media companies develop professional community outreach and education programs to promote control of *Aedes* mosquitoes (*30*). These efforts were associated with higher levels of awareness of control methods and some behavior changes but limited decreases in larval indices (*31*). Rotary International subsequently expanded the program to Colombia, the Philippines, Indonesia, and other countries; the program was the basis for the World Health Organization (WHO) COMBI (communication for behavioral impact) program that is now part of the WHO global strategy for controlling dengue.

During the 1977 epidemic, 76% of municipalities in Puerto Rico instituted public education and cleanup campaigns to reduce mosquito production sites (*9*). Public notification of the epidemic and dengue prevention strategies were conducted through radio and television ads, dissemination of printed materials at public schools, and clinician education; however, the effectiveness of behavioral messaging again could not be evaluated because the epidemic peaked before control measures began (*9*). Evaluation of sustained community education and outreach campaigns conducted during endemic and epidemic DENV transmission during the 1980s and early 1990s demonstrated reasonable success in improving residents’ dengue-related knowledge and reducing mosquito-infested water containers in homes (*31*).

Dengue patient management seminars were frequently offered to clinicians during dengue epidemics starting during the 1970s and focused on case diagnosis and differentiation from other causes of acute febrile illness. As clinical management of dengue patients gradually came into focus as the mainstay secondary method to prevent deaths, in-person clinical training events emphasized the importance of early and appropriate patient management strategies as recommended by WHO (*32*). At the First International Conference on Dengue Hemorrhagic Fever in the Americas hosted by CDC and PRDH in San Juan in 1985, DHF experts from Asia gave plenary lectures at the conference and around the island on all aspects of the disease. A peer education program for physicians and nurses funded by a local pharmaceutical company followed the conference.

As the frequency of clinical trainings waned after the aforementioned changes to the healthcare system in the mid-1990s, case-fatality rates began to rise concurrent with the increasing incidence of dengue and improvements in fatal case detection. Investigation of fatal dengue cases during the 2007 epidemic and a survey of physicians’ practices revealed common missteps in dengue patient management (*33*,*34*). Because similar issues had been observed in Southeast Asia and Central America, the 2009 WHO guidelines for clinical care of dengue patients were incorporated into a 4-hour dengue clinical management course (*35*).

During a large epidemic in 2010, the Puerto Rico Secretary of Health redirected public health resources from vector control campaigns, which had repeatedly been shown to be ineffective, and instead used them to conduct islandwide clinical trainings. To ensure participation, the Secretary made it mandatory for most practicing physicians to complete the course or face a penalty of losing their medical license (*35*). As a result, ≈8,000 clinicians were trained in <6 months, mostly during a 6-week period around the peak of the epidemic. Comparison of physicians’ practice before and after the training demonstrated improvement in several key aspects of dengue clinical case management (*35*). A critical component of the success of these training programs was explaining in detail the rationale behind recommended practices to a cadre of local, well-respected physicians, and having them, not CDC personnel, train their peers throughout the island, as had been done during the 1980s. After the success of the 2010 classroom-based dengue clinical training course, an online training course was also developed (https://www.cdc.gov/dengue/training).

## Advances in Laboratory Diagnostic Testing

**Figure 3:**
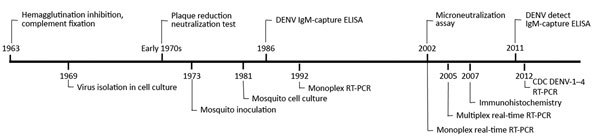
Timeline of incorporation of laboratory techniques used to diagnose suspected dengue reported through the islandwide Passive Dengue Surveillance System in Puerto Rico, 1963–2013. CDC, Centers for Disease Control and Prevention; DENV, dengue virus; RT-PCR, reverse transcription PCR.

The first laboratory diagnostic tool used to detect increased antibody titers in serum specimens collected from persons enrolled in the serosurvey during the 1963 outbreak was hemagglutination inhibition (HI), confirmed with complement fixation (CF) (*6*) (Figure 3). CF also was used during the 1969 serosurvey, and cell culture was used to isolate virus and identify the cause of the 1963 outbreak as DENV-3 (*7*). CF, HI, virus isolation, and plaque-reduction neutralization test (PRNT) were used to test specimens throughout the 1970s (*9*). The first insectary was built in Puerto Rico not to study the mosquito vectors of DENV but to use them as a diagnostic tool. A technique had been developed in Hawaii wherein the serum from suspected dengue patients was intrathoracically injected into *Aedes* or *Toxorhynchites* mosquitoes followed by immunofluorescent detection of DENV antigen (*36*). As case reporting improved during the 1970s, capacity to test all received specimens overwhelmed the system so that only a portion of specimens could be tested.

HI and virus isolation were the most common diagnostic techniques used in the early 1980s to mid-1980s. Use of cultured mosquito cells to isolate virus and immunofluorescence with monoclonal antibodies to identify viruses enabled observation of dissemination of DENV-1 and DENV-4 across Puerto Rico during the 1981–82 epidemic (*19*), as well as the first reported detection of co-infection with 2 DENVs (*37*). During the 1986 epidemic, the IgM antibody capture (MAC) ELISA was adapted to diagnose suspected dengue cases (*38*). MAC-ELISA replaced HI as the standard serologic diagnostic method, enabling simpler diagnosis of patients with suspected dengue. During the first years of the new millennium, CDC participated as a WHO Collaborating Center to evaluate dengue serologic diagnostic tests (*39*) and adapted PRNT for higher throughput by development of the microneutralization assay (*40*). In 2011, the Food and Drug Administration approved a serologic diagnostic test akin to the MAC-ELISA (*41*), and this test is now routinely used in Puerto Rico and elsewhere.

In the early 1990s, CDC developed a reverse transcription PCR (RT-PCR) protocol to detect DENV nucleic acid in serum specimens (*42*). Subsequent RT-PCRs were adapted for real time (rRT-PCR) to assess the magnitude of viremia and for multiplex rRT-PCR to detect all 4 DENVs in the same reaction (*43*,*44*). Automation of RNA extraction began in 2006, and high-throughput RNA amplification was implemented in 2010. Following primer specification to detect a wider variety of modern DENVs, the Food and Drug Administration approved the CDC multiplex rRT-PCR in 2012 (*45*).

**Figure 4:**
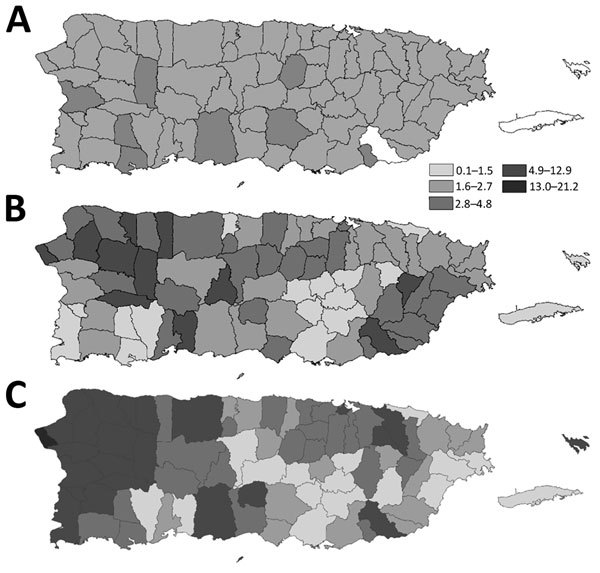
Incidence of laboratory-positive dengue cases reported to Puerto Rico Department of Health by municipality during epidemics in 2007 (A), 2010 (B), and 2012–2013 (C).

rRT-PCR coupled with anti-DENV IgM ELISA became the standard diagnostic tools for diagnosing acute DENV infection, such that the combination of these 2 assays enabled diagnosis of >90% of dengue cases from a single serum specimen (*46*). After improvements to increase laboratory capacity, reported dengue attack rates increased during epidemics during the early 2000s (Figure 4). Diagnosis of fatal dengue cases improved during the 2007 epidemic, when RT-PCR and immunohistochemical analysis were systematically performed on tissue specimens from patients with fatal acute febrile illness (*28*). Contemporary efforts seek to improve the timeliness and utility of dengue diagnostic testing by evaluating point-of-care rapid diagnostic tests (*47*) and modification of molecular assays to simultaneously detect the 4 DENVs, as well as chikungunya and Zika viruses (*48*).

## Development of Tools for Vector Surveillance and Control

Entomologic studies conducted by CDC in Puerto Rico during the early 1960s demonstrated the presence of *Ae. aegypti* mosquitoes in more than half of the homes in neighborhoods affected by dengue (*6*) and these mosquitoes’ susceptibility to various adulticides (*49*). Malathion was used during outbreak responses and during an ultimately unsuccessful *Ae. aegypti* elimination campaign during 1965–1969 (*50*), after which resistance to malathion was identified (reference *51* in Appendix). To attempt to control the 1977 epidemic, space spraying of malathion from trucks and airplanes in San Juan transiently decreased adult mosquito populations, but the number of reported dengue cases did not differ between treated and untreated areas (*9*). Additional studies during the 1970s investigated the environmental determinants of *Aedes* mosquito abundance to show a direct relationship with rainfall and identified discarded tires and animal water pans as common breeding sites (references *52*,*53* in Appendix).

Extensive research was conducted during the 1980s on use of insecticides to control adult *Ae. aegypti* densities; use of larvicides (e.g., temephos, *Bacillus thuringiensis*), biocontrol (i.e., copepods), and source reduction to control larvae; and studies of vector competence and transmission dynamics. The efficacy of ultralow volume space spraying with malathion and pyrethroids was evaluated in 1987, as was aerial application of naled using C-130 aircraft. Unfortunately, such efforts had no effect on epidemic trends and variable levels of success in reducing DENV transmission (*31*).

After having been standardized as a vector surveillance tool during the *Ae. aegypti* eradication program during the 1960s (*50*), the ovitrap was modernized at the CDC Dengue Branch during the 1980s (reference *54* in Appendix). Around the same time, the backpack mosquito aspirator was adapted for adult mosquito collection in households (reference *55* in Appendix), enabling direct quantitation of the absolute number of mosquitoes in a household. These methods were also used in place of bioassays to evaluate the efficacy of space spraying.

After observations that adult mosquito populations do not correlate well with larval densities, in the early 2000s pupal populations were reported to have a nonlinear relationship with density of adults (reference *56* in Appendix). As had been done at the US Department of Agriculture, vector surveys were simplified to focus on pupae as a predictor of vector abundance (reference *57* in Appendix). The focus on pupae for vector surveys in turn confirmed that if mosquito surveillance monitors only immature mosquitoes, no effect on adult populations is detected after vector control interventions, as had been observed by others. Later efforts demonstrated an association between household densities of adult female *Ae. aegypti* mosquitoes and risk for DENV infection (reference *58* in Appendix), and cryptic breeding sites (e.g., septic tanks) were identified as major producers of adult mosquitoes (reference *59* in Appendix). Contemporary efforts have focused on the design of autocidal gravid ovitraps that can be used for simultaneous mosquito surveillance and control and are associated with sustainable decreases in vector abundance (reference *60* in Appendix) and reduced risk for infection from pathogens transmitted by *Ae. aegypti* mosquitoes (reference *61* in Appendix). Evaluations of autocidal gravid ovitraps on a larger scale are under way.

## Conclusions

Dengue remains a major public health concern throughout the tropics and subtropics. In Puerto Rico, close alliance of CDC with PRDH has proven to be integral not only in detecting and responding to epidemics but also in furthering the collective understanding of the molecular, diagnostic, epidemiologic, and entomologic characteristics of dengue. Dengue surveillance and research have therefore demonstrated a mutually beneficial and interdependent relationship to combat dengue. Dengue surveillance also has promoted the recognition and study of nondengue acute febrile illnesses, an attribute further shown during the recent emergence in Puerto Rico of chikungunya virus in 2014 and Zika virus in 2015 (references *62*,*63* in Appendix).

The demonstrated limitations in chemical approaches to dengue control have inspired several alternative interventions (reference *64* in Appendix). As dengue vaccines and vector control interventions continue to be developed and evaluated, the need for surveillance and research to design, implement, and evaluate these tools will continue. Academic, public, and private organizations play both complementary and overlapping roles in various aspects of dengue surveillance and research; thus, close partnerships will continue to be integral components of successful public health initiatives to combat dengue. Because of decades of experience and baseline surveillance data, Puerto Rico is expected to continue to be a site that leads evaluation of interventions designed to control dengue. Collaborations such as that of CDC, the Pan American Health Organization, WHO, and PRDH will be instrumental in such efforts, as will implementation of lessons learned from Puerto Rico and other areas.

Appendix Additional references for lessons learned from dengue surveillance and research, Puerto Rico, 1899–2013.
