# Supplementary material for: Lessons Learned from Dengue Surveillance and Research, Puerto Rico, 1899–2013
Source: Emerg Infect Dis. 2019 Aug;25(8):1522–30. doi: 10.3201/eid2508.190089 (PMC6649339; doi:10.3201/eid2508.190089)
Supplement: Appendix — Additional references for lessons learned from dengue surveillance and research, Puerto Rico, 1899–2013. [file 19-0089-Techapp-s1.pdf]

Article DOI: https://doi.org/10.3201/eid2508.190089

Lessons Learned from Dengue Surveillance and Research, Puerto Rico, 1899–2013

Appendix

Additional References

51. Fox I, Bayona IG. Malathion resistant strains of *Aedes aegypti* in Puerto Rico in 1969. Mosq News. 1972;32:157–60.

52. Moore CG, Cline BL, Ruiz-Tibén E, Lee D, Romney-Joseph H, Rivera-Correa E. *Aedes aegypti* in Puerto Rico: environmental determinants of larval abundance and relation to dengue virus transmission. Am J Trop Med Hyg. 1978;27:1225–31. PubMed http://dx.doi.org/10.4269/ajtmh.1978.27.1225

53. Lee DM. C.G. Mosquito studies during an interepidemic outbreak of dengue in Puerto Rico. Mosq News. 1973;33:506–9.

54. Reiter P, Amador MA, Colon N. Enhancement of the CDC ovitrap with hay infusions for daily monitoring of *Aedes aegypti* populations. J Am Mosq Control Assoc. 1991;7:52–5. PubMed

55. Clark GG, Seda H, Gubler DJ. Use of the "CDC backpack aspirator" for surveillance of *Aedes aegypti* in San Juan, Puerto Rico. J Am Mosq Control Assoc. 1994;10:119–24. PubMed

56. Barrera R, Amador M, Clark GG. Use of the pupal survey technique for measuring *Aedes aegypti* (Diptera: Culicidae) productivity in Puerto Rico. Am J Trop Med Hyg. 2006;74:290–302. PubMed http://dx.doi.org/10.4269/ajtmh.2006.74.290

57. Barrera R. Simplified pupal surveys of *Aedes aegypti* (L.) for entomologic surveillance and dengue control. Am J Trop Med Hyg. 2009;81:100–7. PubMed http://dx.doi.org/10.4269/ajtmh.2009.81.100

58. Rodriguez-Figueroa L, Rigau-Perez JG, Suarez EL, Reiter P. Risk factors for dengue infection during an outbreak in Yanes, Puerto Rico in 1991. Am J Trop Med Hyg. 1995;52:496–502. PubMed http://dx.doi.org/10.4269/ajtmh.1995.52.496

59. Barrera R, Amador M, Diaz A, Smith J, Munoz-Jordan JL, Rosario Y. Unusual productivity of *Aedes aegypti* in septic tanks and its implications for dengue control. Med Vet Entomol. 2008;22:62–9. PubMed http://dx.doi.org/10.1111/j.1365-2915.2008.00720.x

60. Barrera R, Amador M, Acevedo V, Hemme RR, Félix G. Sustained, area-wide control of *Aedes aegypti* using CDC autocidal gravid ovitraps. Am J Trop Med Hyg. 2014;91:1269–76. PubMed http://dx.doi.org/10.4269/ajtmh.14-0426

61. Lorenzi OD, Major C, Acevedo V, Perez-Padilla J, Rivera A, Biggerstaff BJ, et al. Reduced incidence of chikungunya virus infection in communities with ongoing *Aedes Aegypti* mosquito trap intervention studies—Salinas and Guayama, Puerto Rico, November 2015–February 2016. MMWR Morb Mortal Wkly Rep. 2016;65:479–80. PubMed http://dx.doi.org/10.15585/mmwr.mm6518e3

62. Adams L, Bello-Pagan M, Lozier M, Ryff KR, Espinet C, Torres J, et al. Update: ongoing Zika virus transmission—Puerto Rico, November 1, 2015–July 7, 2016. MMWR Morb Mortal Wkly Rep. 2016;65:774–9. PubMed http://dx.doi.org/10.15585/mmwr.mm6530e1

63. Sharp TM, Ryff KR, Alvarado L, Shieh WJ, Zaki SR, Margolis HS, et al. Surveillance for chikungunya and dengue during the first year of chikungunya virus circulation in Puerto Rico. J Infect Dis. 2016;214(suppl 5):S475–81. PubMed http://dx.doi.org/10.1093/infdis/jiw245

64. Achee NL, Grieco JP, Vatandoost H, Seixas G, Pinto J, Ching-Ng L, et al. Alternative strategies for mosquito-borne arbovirus control. [Erratum in: PLoS Negl Trop Dis. 2019. PLoS Negl Trop Dis. 2019;13:e0006822. PubMed http://dx.doi.org/10.1371/journal.pntd.0006822
